# Recycling of Silicomanganese Slag and Fly Ash for Preparation of Environment-Friendly Foamed Ceramics

**DOI:** 10.3390/ma16206724

**Published:** 2023-10-17

**Authors:** Guihang Yu, Wei Gao, Yanbin Yao, Tingting Zhang, Ying Fu, Xiangqing Kong

**Affiliations:** 1 School of Civil Engineering, Liaoning University of Technology, Jinzhou 121001, China; guihang7900@163.com (G.Y.); 15684170962@163.com (W.G.); lgyaoyanbin@163.com (Y.Y.); zhangtt235@163.com (T.Z.); 2 Songshan Lake Material Laboratory, Dongguan 523808, China; 3 College of Pipeline and Civil Engineering, China University of Petroleum (East China), Qingdao 266580, China

**Keywords:** foamed ceramics, silicomanganese slag, fly ash, anorthite, sintering temperature

## Abstract

In order to reduce the manufacturing cost of foamed ceramics and expand the application scope of industrial solid waste, in this study, a new type of environment-friendly foamed ceramics was prepared using direct high-temperature foaming with waste silicomanganese slag (SMS) and fly ash (FA) as raw materials and silicon carbide (SiC) as a foaming agent. The influence of SMS content, SiC content, and sintering temperature on the characteristics and microstructure of the specimen were explored. More concretely, the compressive strength, pore morphology, bulk density, and crystalline composition of the foamed ceramics were discussed. The foaming mechanism was also further analyzed. The results showed that including 20% SMS significantly reduced the melt’s viscosity and stimulated bubble expansion. This, in turn, facilitated the creation of a porous structure. Moreover, it was noted that samples containing 20% SMS exhibited an anorthite phase when sintered at 1110 °C, resulting in enhanced compressive strength. The bulk density and compressive strength of the foamed ceramics decreased with an increase in the sintering temperature and SiC content. This trend was primarily attributed to the higher total porosity and the insufficient support of the pore wall to the matrix. The best all-around performance was achieved with 20 wt% SMS, 80 wt% FA as raw material, SiC addition of 1.0 wt%, and a sintering temperature of 1100 °C. Under these conditions, the compressive strength, bulk density, and total porosity of the foamed ceramics were 8.09 MPa, 0.57 g/cm^3^, and 71.04%, respectively. Taken together, the outstanding porous structure and mechanical properties of this foamed ceramic make it suitable for use as insulation or for building partition materials.

## 1. Introduction

Foamed ceramics are a new porous material that has received worldwide attention due to their unique lightweight, sound insulation, and heat preservation properties. These properties make foamed ceramics widely used in heat insulation, sound-absorption, and lightweight construction materials [1,2,3]. Foamed ceramics are mainly made of clay, quartz, or silica micropowder and alumina powder as the main raw material, with the addition of a foaming agent that is mixed well and foamed at a high temperature [4,5,6,7]. The manufacture of foamed ceramics consumes large amounts of ceramic and chemical materials. The high production costs impose limitations on the sustainability of the ceramics industry.

Extensive research has been conducted on using natural minerals as substitutes for traditional raw materials in ceramics production to achieve cost reduction [8]. Sumi et al. [9] successfully prepared dense cordierite ceramics using kaolinite at 1350 °C. Likewise, Goren et al. [10] achieved the successful synthesis of cordierite ceramics at 1350 °C by incorporating talc as a raw material component. Banjuraizah et al. [11] synthesized cordierite glass–ceramic from talc, kaolinite, and a small amount of reagent-grade oxide. These studies indicate that natural minerals are a high-quality substitute for conventional raw ceramic material production.

Nonetheless, Wang et al. [12] expressed apprehensions regarding the excessive depletion of natural minerals caused by the growing ceramic product demand. Consequently, numerous researchers have turned to utilising solid wastes rich in Si and Al as alternatives to natural minerals. Liu et al. [13]. achieved the successful preparation of porous cordierite ceramics utilizing ferrochrome slag. However, heavy metal chromium within the structure restricts the extensive use of these products. Yio et al. [14] reported the preparation of foamed glass-ceramics from furnace bottom ash. Wu et al. [15] employed steel slag in the production of porous ceramics. However, the process of grinding steel slag led to an escalation in the manufacturing expenses of the porous ceramics. Aside from the previously mentioned solid wastes, fly ash (FA) becomes a promising candidate to substitute ceramic raw materials [16].

FA, a by-product of coal combustion, is one of the world’s most emitted solid wastes. The amount of FA generated in China is 686 million tons annually [17]. Such a great amount of FA has resulted in serious environmental issues, including air, soil, and groundwater contamination. Hence, there is an urgent need to find a beneficial means to treat FA to boost recovery rates and reduce its contamination. In recent years, FA has been widely used to prepare ceramics due to the abundance of Al_2_O_3_ and SiO_2_ [18,19,20]. Liang et al. [21] successfully synthesized foamed ceramics using FA and ceramic tile polishing waste at 925 °C. They concluded that the foamed ceramics obtained from 25 wt% FA and 75 wt% ceramic polishing waste had excellent mechanical properties (7.8 MPa). Wang et al. [22] used high alumina FA to fabricate bi-layered foam glass ceramic tile for building insulation. The bending strength of this bi-layered ceramic tile was observed to reach 31.5 MPa after sintering at 1200 °C. Furthermore, the high alumina FA used in the specimens could obtain more than 70 wt%. Huo et al. [23] achieved the first successful preparation of mullite foamed ceramics using fly ash hollow spheres as raw materials. The results revealed that, after sintering at 1350 °C, the samples demonstrated a remarkable porosity of 81% and a substantial compressive strength of 33.4 MPa. Likewise, Yin et al. [24] fabricated cost-effective and highly efficient porous SiC-Al_2_O_3_ ceramics by employing fly ash as the primary raw material.

As a raw material substitute for foamed ceramics, another solid waste to be noted is silico–manganese slag (SMS), which is an industrial by-product of silico–manganese ore reduced by lime and coke at high temperature. The output of SMS in China in 2019 was as high as 1.25~13.585 million tons [25]. Previous studies have suggested that SiO_2_ and Al_2_O_3_ in SMS are considerable, as are fluxes such as Na_2_O, K_2_O, etc., which are favorable to producing a molten glass phase at the same temperature [26]. In addition, the appropriate amount of CaO in SMS can improve the compressive strength of the material due to the crystalline phase of anorthite increasing with the CaO content [27]. With these benefits, SMS has excellent potential as a raw material for foamed ceramics. However, SMS currently produces mainly low value-added construction materials such as cement [28,29,30], concrete [31], alkali-activated binders [32,33], etc. In the existing research work, few studies have been found on the use of SMS to prepare foam ceramics. Consequently, an in-depth investigation is necessary to understand the effect of SMS on the properties of foamed ceramics. This paper explores the feasibility of producing foamed ceramics from FA and SMS, especially the SMS content, which will be investigated to address the SMS gap in the foamed ceramics field.

The primary aim of this study is to efficiently utilize FA and SMS in foamed ceramics materials for cost reduction. The influences of the SMS content and sintering temperature on the bulk density, compressive strength, pore morphology, crystalline phase composition, and microstructure of foamed ceramics were systematically investigated. Additionally, the effects of the SiC content on the properties and pore structure of foamed ceramics was discussed. The foaming mechanism was also further analyzed. This work widely guides utilizing solid waste in the industry, with significant implications for environmental conservation and resource recycling.

## 2. Materials and Methods

### 2.1. Materials and Reagents

The FA was from the Zhengzhou power plant in Henan, China. The SMS was obtained from the Jinzhou steel plant in Liaoning, China. The foaming agent silicon carbide (SiC) was obtained from Keze Metal Materials Co., Ltd. in Xingtai, China. Borax and bentonite are from Jinzhou, Liaoning Province, China. Borax (Na_2_B_4_O_5_(OH)·10H_2_O) was used as a flux to lower the softening temperature of the billet. Bentonite acted as the binder to ensure the strength of the billet after drying.

Table 1 shows the chemical compositions of FA and SMS using an X-ray fluorescence spectrometer (XRF). Silicon and aluminum were the principal chemical elements in the FA and SMS. Small amounts of calcium and magnesium were also detected in the SMS. There were approximately 20 other elements in the FA and SMS, e.g., V_2_O_5_, Cr_2_O_3_, Co_3_O_4_, ZnO, CuO, etc. However, V_2_O_5_ accounted for the most significant proportion of the other components, only 0.063%; therefore, the influence of the other components on the product can be considered negligible.

**Figure 1:**
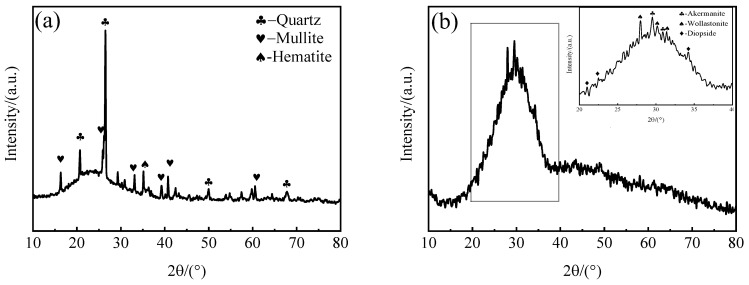
XRD patterns of the FA and SMS. (**a**) FA (**b**) SMS.

The mineralogical composition of FA and SMS were researched using X-ray diffraction (XRD) (Figure 1). As can be seen in Figure 1a, there is a distinct hump approximately between 2*θ* = 15° and 35°. This was owing to the presence of several amorphous glass phases in the FA. The primary mineral types in the FA were quartz (PDF# 78-1253, SiO_2_), mullite (PDF# 15-0776, Al_6_Si_2_O_13_), and hematite (PDF# 72-0469, Fe_2_O_3_). In Figure 1b, a prominent “bun-like peak” can be observed, indicating that the SMS mainly existed in the form of an amorphous phase, although some diffraction peaks exist at 20–40°(2*θ*). The primary mineral types in the SMS were quartz (PDF# 85-0865, SiO_2_), wollastonite (PDF# 29-0372, CaSiO_3_), diopside (PDF# 75-1577, CaMg(SiO_3_)_2_) and Åkermanite (PDF# 35-0592, Ca_2_MgSi_2_O_7_).

### 2.2. Material Treatment and Sample Preparation Processes

**Figure 2:**
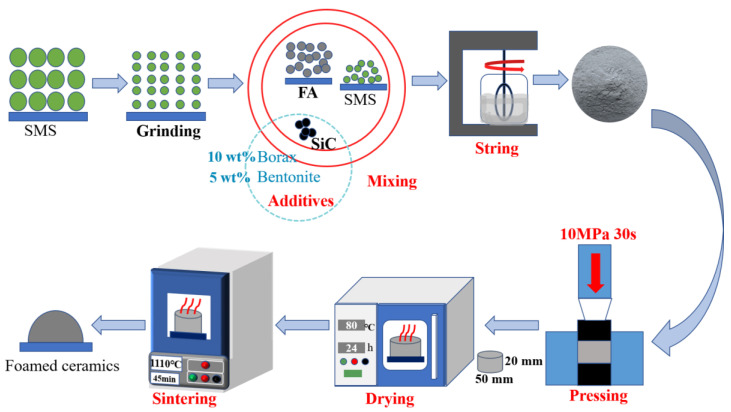
Schematic illustration of the foamed ceramics preparation process.

Figure 2 shows the fabrication procedure of the foamed ceramics. The SMS was ball-milled for 4 h to homogenize and was sieved through an 80 μm sieve after drying. Then, the FA and SMS were mixed with different components, with 10 wt% borax as the flux and 5.0 wt% bentonite as the binder. Meanwhile, 0~3.0 wt% SiC was added as a foaming agent. Table 2 shows the components of all the samples. The mixture was then thoroughly blended with a specific quality of water. An electric tablet was pressed under 10 MPa for 30 s into green billets with a radius and length of 25 mm and 20 mm, respectively. The green billets were dried in an oven until the weight remained stable. The dried billets were sintered at the desired temperature for 45 min at a heating speed of 300 °C/h in a heating furnace.

### 2.3. Characterization

#### 2.3.1. X-ray Diffraction (XRD)

XRD (Bruker-axs D8 Advance, Germany) was employed to analyze the mineral ingredients of the raw material and track the changes in the crystal of the product. The scanning rate was set at 2°/min within the range of 10~80° using CuKα radiation.

#### 2.3.2. Scanning Electron Microscopy (SEM)

The samples’ microstructures were examined using a scanning electron microscope (SEM) of type SU-8010 from JEOL, Japan. Prior to the SEM experiments, the foamed ceramics were precisely sectioned into small pieces meeting the inspection criteria. Subsequently, they were sprayed with platinum using a sputter coater.

#### 2.3.3. Pore Size Analysis

Sample photographs were captured using a Sony Alpha 7R IV (Amazon.com, Inc., Washington, DC, USA) full-frame mirrorless digital camera to observe the pore morphology of the samples. Subsequently, 100 randomly chosen pores from the sample photos were analyzed for pore size using Nano-measure 1.2 software.

### 2.4. Physical and Mechanical Property Testing

The bulk density and apparent porosity of the sintered samples were identified using the Archimedes method using Equations (1) and (2). Firstly, the samples were dried to a constant weight and weighed to determine their dry weight, *m*_1_. After the sample was placed in water, the water was boiled for 2 h. Then, the water was naturally cooled to room temperature, at which time *m*_2_ was the mass of the specimen in water. Finally, the surface water of the sample was wiped with a wet towel, and the damp weight *m*_3_ was recorded. Equation (3) evaluates the total porosity. The closed porosity was calculated using Equation (4):

(1) $$\rho_{b}=\frac{m_{1}}{\left(m_{3}-m_{2}\right)}$$

(2) $$V_{open}=\frac{\left(m_{3}-m_{1}\right)}{\left(m_{3}-m_{2}\right)}\times 100\%$$

(3) $$V_{p}=\left(1-\frac{\rho_{b}}{\rho_{t}}\right)\times 100\%$$

(4) $$P_{closed}=V_{p}-V_{open}$$

where *ρ_b_* is the bulk density of the sintered samples, *V_open_* and *V_p_* are the apparent and total porosities of the sintered samples, respectively, *P_closed_* is the closed porosity of the sintered samples, and *ρ_t_* is the true density of the sintered samples.

The compression test was conducted using the WDW300 electronic universal testing machine (Jinan Hensgrand Instrument Co., Ltd., Jinan, China) based on the Chinese standard (GB/T 1964-1996 [34]). A 1 mm/min loading rate was applied, and the compressive strength was determined by recording the maximum load during the loading process. To ensure the reliability of the experimental results, six samples were selected for testing for each performance parameter, and the average value was subsequently calculated. All performance measurements are accurate to 3 decimal places.

## 3. Results and Discussion

### 3.1. The Influence of SMS Content on the Properties of Foamed Ceramics

#### 3.1.1. Effect of the SMS Content on the Physical and Mechanical Properties

**Figure 3:**
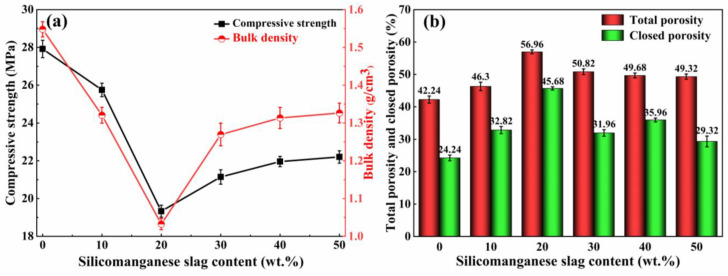
Effect of the silicomanganese slag content on the physical and mechanical properties of the samples. (**a**) Compressive strength and bulk density; (**b**) total porosity and closed porosity.

Figure 3a presents the influence of the SMS content on the characteristics of the foamed ceramics. With the addition of SMS, the bulk density of the product first decreased and then increased, following the same trend as the compressive strength. When the SMS content is varied from 0 to 20 wt%, the bulk density of the samples decreased dramatically from 1.55 g/cm^3^ to a minimum value of 1.03 g/cm^3^, and the compressive strength also decreased from 27.91 MPa to a minimum value of 19.33 MPa. As shown in Figure 3b, the total porosity and closed porosity increased from 42.24% and 24.24% to the maximum values of 56.96% and 45.68%, respectively, when the SMS content increased from 0 to 20 wt%. There are several reasons for this decrease in density and strength. The addition of SMS introduces additional CaO and MgO, which effectively disrupts the Si-O-Si linkages within the silicate network structure, reducing the strength of [SiO_4_] tetrahedra [35]. In the meantime, the degree of polymerization (DOP) of the old silicate network is reduced, resulting in a decrease in the liquid viscosity [36]. Expansion resistance to pore growth decreases with decreasing liquid viscosity. Therefore, the pores inside the foamed ceramic increase, and the bulk density and compressive strength decrease. Additionally, Na_2_O and K_2_O influence the samples’ bulk densities and compressive strengths. These elements reduce the melting point of the mixture and promote the formation of a molten glass phase during the sintering process [37,38]. At a 20 wt% SMS content, the glassy phase liquid encapsulates gas, forming numerous bubbles. This leads to an increase in the total and closed porosity while causing a reduction in the bulk density and compressive strength. This phenomenon shows a negative correlation trend between bulk density, compressive strength, and porosity. Ge et al. [39] consistently observed that as the bulk density of coal bottom ash foamed ceramics decreased from 0.80 g/cm^3^ to 0.20 g/cm^3^, the compressive strength decreased from 37.65 MPa to 2.05 MPa. However, the porosity increased from 68.4% to 87.5%. As the content of the SMS exceeded 20 wt%, the liquid viscosity decreased further, and the molten glass phase was insufficient to encapsulate the internal pores of the sample. This led to porous shared pore walls and connected pores, reducing the total and closed porosity and increasing the bulk density and compressive strength. Furthermore, the increase in CaO promoted the formation of anorthite, which enhanced the compressive strength. Nonetheless, an excess of anorthite hindered the foaming process.

#### 3.1.2. Effects of the SMS Content on the Pore Structure

**Figure 4:**
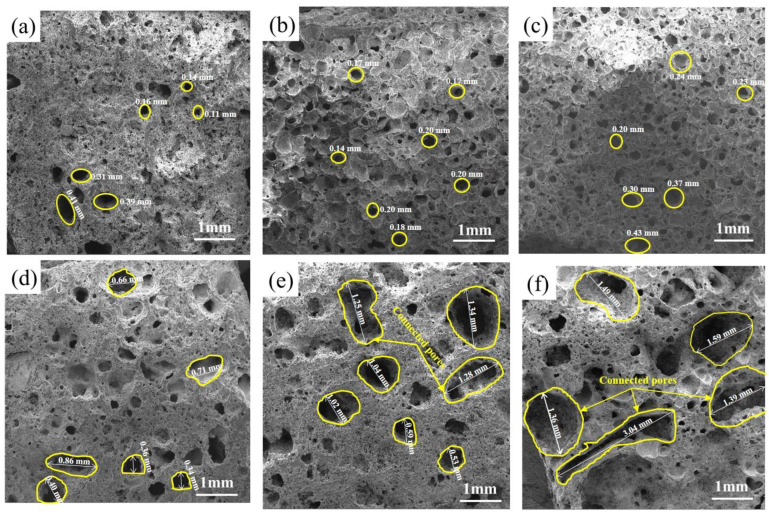
Pore morphology of samples with different silicomanganese slag content. (**a**) 0 wt%, (**b**) 10 wt%, (**c**) 20 wt%, (**d**) 30 wt%, (**e**) 40 wt%, (**f**) 50 wt%.

Figure 4 presents the pore morphology of the different SMS specimens, with the pores increasing in size with increased SMS content. It can be observed from Figure 4a that the pores of the specimens without SMS were smaller. When the SMS content was increased from 0 to 20 wt%, the pore size is increased gradually, and the total porosity increased continuously. CaO and MgO reduced the viscosity of the molten glass phase, thus diminishing its resistance to bubble expansion. On the flip side, the presence of Na_2_O and K_2_O facilitates the generation of more molten glass phase. The growing amount of gas is encapsulated by the molten glass phase and expands rapidly. The amount of pores is significantly reduced, and cross-linking or fusion occurs between the bubbles in Figure 4d. This may be due to the further increase in SMS content and the continued decrease in liquid viscosity, which worsens the effects of the molten glass phase in wrapping pores during sintering. When SMS is added at 50 wt%, the pore structure deteriorates further, and large connected pores appear. The too-low viscosity of the molten glass phase can explain the phenomenon in Figure 4f. The low viscosity of the liquid prevents it from enclosing individual bubbles, resulting in the formation of interconnected pores with shared walls.

#### 3.1.3. Effects of SMS on the Crystalline Phase

**Figure 5:**
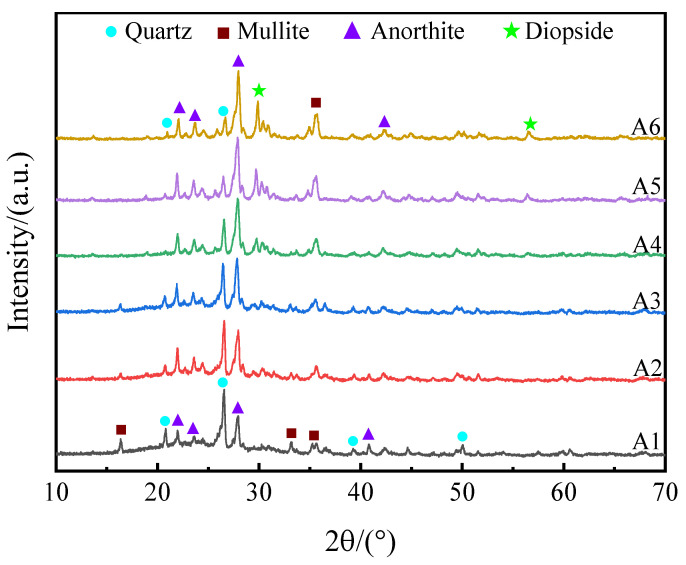
XRD patterns of samples with different silicomanganese slag contents.

**Figure 6:**
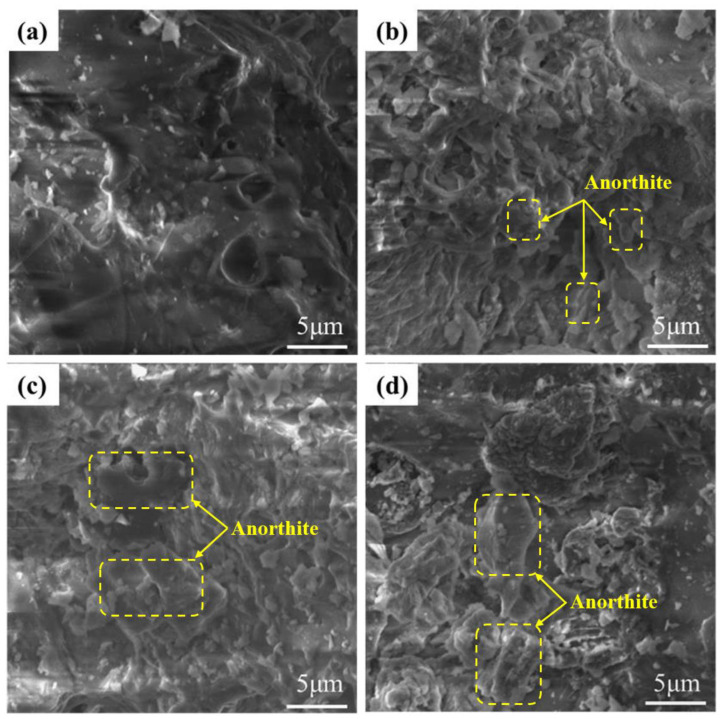
Microstructure of samples with different silicomanganese slag contents. (**a**) 0%, (**b**) 20%, (**c**) 30%, (**d**) 50%.

Figure 5 presents the mineral phases of specimens A1–A6. The primary mineral composition of specimens A1–A6 were quartz (PDF#79-1906, SiO_2_), mullite (PDF#15-0776, Al_6_Si_2_O_13_), anorthite (PDF#89-1471, Ca(Al_2_Si_2_O_8_)), and diopside (PDF#72-1497, CaMgSi_2_O_6_). With the addition of SMS, the number of anorthite crystals increased, and the amount of the quartz phase decreased inch by inch (A1–A3). This is mainly attributed to the combined action of Al^3+^ and Ca^2+^ replacing Si^4+^, which destroys the original quartz phase and promotes the formation of anorthite [40]. At this time, the increase in anorthite has a weaker effect on the growth of pores, and CaO and MgO contribute a significant part to the depolymerization of the silicate network. The content of anorthite continues to increase (A4–A6), decreasing the molten glass phase. The total porosity rate reduces when the liquid phase content decreases to the extent that it is insufficient to encapsulate the growing pore completely. The porosity change tendencies corresponded in Figure 3b as well. In addition, it is clearly observed in Figure 6 that the columnar and lamellar anorthite gradually increases on the pore wall with increasing SMS content. A faint diopside phase was first observed in sample A4. The incorporation of SMS leads to an accompanying increase in diopside content, thereby contributing to a gradual enhancement in compressive strength [41,42]. However, excessive anorthite was observed to diminish the ability to generate a liquid phase, which adversely affects product foaming.

### 3.2. The Influence of Sintering Temperature on the Properties of Foamed Ceramics

#### 3.2.1. Effects of the Sintering Temperature on the Physical and Mechanical Properties

**Figure 7:**
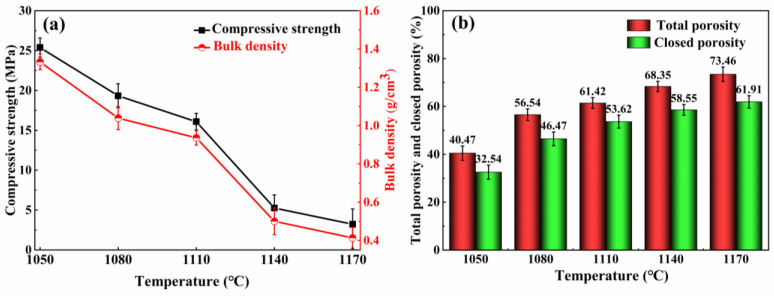
Effects of sintering temperature on the mechanical and physical properties of the samples. (**a**) Compressive strength and bulk density, (**b**) total porosity and closed porosity.

The firing temperature has a significant impact on the characteristics of the burnt material. In this section, the influence of temperature on the foamed ceramics was investigated using the A3 specimens. Figure 7 illustrates the physical and mechanical properties of the specimens at various sintering temperatures. As the temperature ranges from 1050 °C to 1110 °C, the compressive strength of the specimens decreased gradually from 25.38 MPa to 16.08 MPa, and the bulk density followed a similar trend, decreasing from 1.33 g/cm^3^ to 0.94 g/cm^3^ in Figure 7a. The compressive strength of the foamed ceramics decreased significantly, from 16.08 MPa to 5.25 MPa, representing a reduction of approximately 67.4%, when sintered at temperatures ranging from 1110 °C to 1140 °C. The sharp decrease in compressive strength was detrimental to the stability of the foam ceramics. When the temperature was elevated to 1170 °C, the bulk density and compressive strength decreased to a minimum of 0.41 g/cm^3^ and 3.23 MPa. The variation patterns in the total and closed porosity at various firing temperatures is shown in Figure 7b. The total and closed porosities exhibited a continuous increment with the increased firing temperature. The total porosity peaked at 1170 °C, reaching a value of 73.46%.

Based on previous studies [43,44], a compact SiO_2_ protective membrane on the SiC surface acts as a diffusion barrier, thus slowing down the SiC oxidation. The molten glass phase increases with the firing temperature from 1050 °C to 1100 °C. The SiO_2_ protective layer easily forms a silicate liquid phase with alkaline molten salt under high-temperature conditions, which in turn leads to the corrosion and destruction of the SiO_2_ protective layer and accelerates the reaction between SiC and O_2_, resulting in a large amount of CO and CO_2_ gas production [45]. The produced gas is encapsulated in the porcelain body by the liquid phase, and closed pores are generated, explaining the decreased compressive strength and bulk density. On the other hand, at high sintering temperatures, SiC continues to react with oxygen to produce more gas. The inner pressure of the bubble increases, expanding the pore size and causing a thinning of the pore wall, which leads to an increase in the total porosity of the foamed ceramics. Subsequently, the effective viscosity of the liquid decreases further with increasing temperature (>1110 °C). The pore wall cannot maintain the air pressure inside the pore, and the pores merge. With the appearance of merging pores, the pore diameter further increases, the pore wall thickness continues to decrease, and the large pores rupture and collapse, leading to the collapse of the porcelain body. This is the reason for the dramatic decrease in compressive strength and bulk density.

#### 3.2.2. Effect of the Sintering Temperature on the Pore Structure

**Figure 8:**
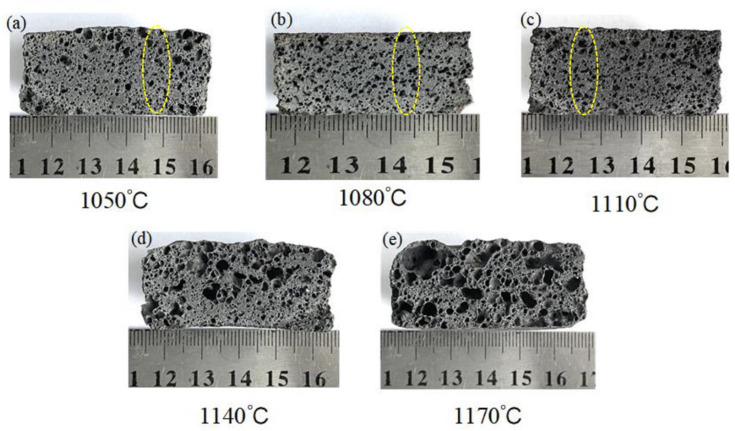
The cross-section macrostructure of the samples at different temperatures.

The cross-sectional morphology of the specimens at various sintering temperatures is shown in Figure 8. The samples at different sintering temperatures exhibited other characteristics, including changes in pore size, pore wall thickness, and pore morphology. As the sintering temperature increased (1050~1110 °C), the number of pores increased, and the pore sizes became larger. At high temperatures, more SiC reacts, producing more gas [46]. On the other hand, the viscosity of the molten liquid reduces inch by inch as the firing temperature increases, facilitating the expansion of the pore size [47]. In Figure 8d,e, when the sintering temperature is too high (≥1140 °C) and the molten phase viscosity is excessively low, merging and bridging between bubbles occurs, leading to an increase in interconnected pores. This increase is also responsible for the significant decrease in the compressive strength and bulk density. The gas produced during the sintering process follows the ideal gas law equation given in Equation (5):

(5) PV=nRT

where *P* is the gas pressure, *V* is the pore volume, *n* is the amount of gas substance, *R* is the gas constant, and *T* is the temperature. As the temperature increases, the pore volume and total porosity gradually increase, which is consistent with the conclusion drawn in Figure 7. In addition, the pores grew more prominent from the bottom up, inch by inch, as marked by the circles in Figure 8. Other conditions being equal, the decrease in the melt viscosity accelerates the rate of bubble rise [48]. As the temperature increases, the smaller pores dissolve between the larger pores, increasing the probability of the bubbles combining. This phenomenon satisfies the Stokes formula in Equation (6):

(6) $$v=(2(\rho_{1}-\rho_{2})gr^{2})/(9\eta )$$

where ν is the pore rising velocity, *ρ*_1_ and *ρ*_2_ are the densities of high-temperature liquid and gas in the pore, respectively, *r* is the pore radius, *η* is the effective viscosity, and *g* is the acceleration due to gravity.

**Figure 9:**
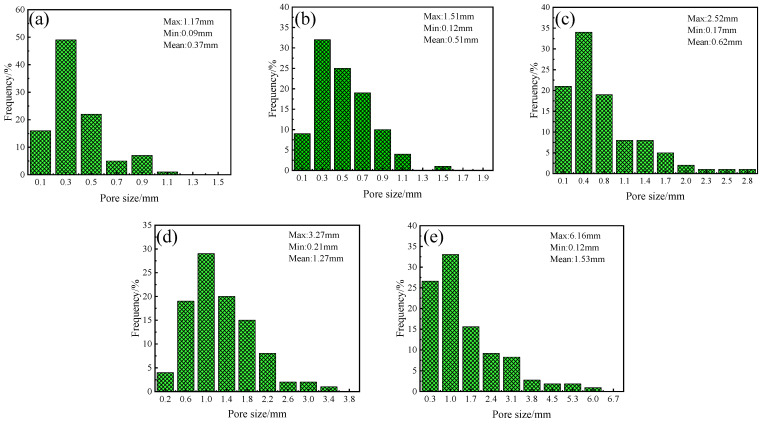
Pore size distribution of samples at different sintered temperatures. (**a**) 1050 °C, (**b**) 1080 °C, (**c**) 1110 °C, (**d**) 1140 °C, (**e**) 1170 °C.

Figure 9 depicts the pore diameter range for sample A3 at 1050~1170 °C. The average pore diameters of the sintered samples were 0.37 mm, 0.51 mm, 0.62 mm, 1.27 mm, and 1.53 mm at 1050~1170 °C. Figure 9d presents large pores with diameters larger than 3 mm and very uneven distributions. When the temperature was 1170 °C, the distribution of the pores deteriorated sharply, and oversized pores with a diameter of 6.16 mm appeared in the samples. The samples sintered at 1050 °C, 1080 °C, and 1110 °C had a relatively homogeneous pore size distribution. However, the maximum pore size in Figure 9a,b was only 1.51 mm, indicating that the samples’ foaming was insufficient at 1050 °C and 1080 °C. The above results show that the sintering temperature dramatically influences the pore diameter and distribution of the specimens, and that 1110 °C is the optimum firing temperature. In summary, changing the firing temperature allows for effective control of the pore structure of foamed ceramics.

#### 3.2.3. Effects of Sintering Temperature on the Crystalline Phase

**Figure 10:**
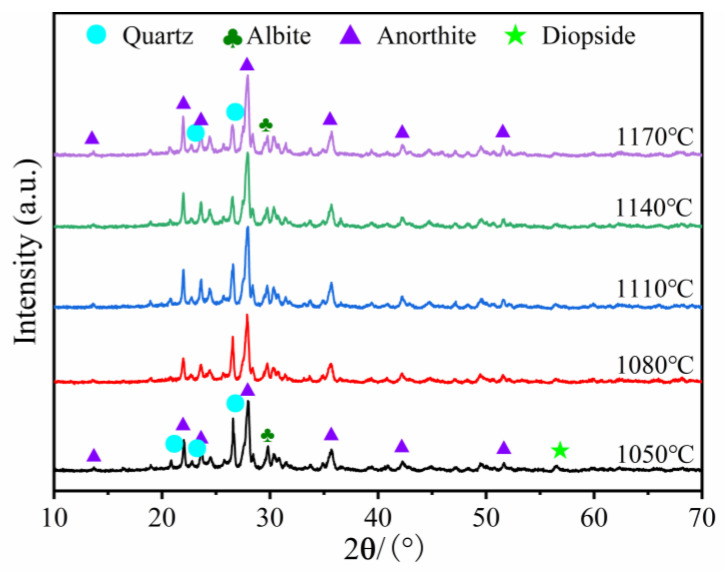
XRD patterns of samples sintered at different temperatures.

The influence of temperature on the mineral phase of the A3 product is presented in Figure 10. The major mineral composition of sample included quartz (PDF#79-1906, SiO_2_), Mullite (PDF#15-0776, Al_6_Si_2_O_13_), anorthite (PDF#89-1471, Ca(Al_2_Si_2_O_8_)), and diopside (PDF#72-1497, CaMgSi_2_O_6_). The firing temperature ranged from 1050 °C to 1170 °C, and the decrease in the quartz phase in the sintered product was not accompanied by the formation of new mineral phases. Close inspection revealed that the amorphous bulge in the 2*θ* = 15~35° span steadily increased. This was due to the increased temperature, causing larger quantities of quartz to melt and form a glassy phase. The formation of the glass phase provided a sufficient liquid phase for the foaming process and promoted an increase in the total porosity. The presence of quartz increased the specimen’s compressive strength but decreased the product’s liquid phase generation capacity at high temperatures, which inhibited sample foaming. Wang et al. [36] demonstrated that adding Al_2_O_3_ powder to porous ceramics reduces the liquid phase generation capacity of the specimen, which increases the quartz crystalline content and promotes the product’s mechanical properties. In the range of 1050 °C to 1170 °C, the mullite and diopside peak intensities declined somewhat, whereas the anorthite peak intensities remained nearly constant, as the melting point of anorthite is approximately 1530 °C. Moreover, the presence of anorthite ensures that the compressive strength of the foamed ceramics will not be too low.

### 3.3. The Influence of Foaming Agent Content on the Properties of Foamed Ceramics

#### 3.3.1. Effects of the Foaming Agent Content on the Physical and Mechanical Properties

**Figure 11:**
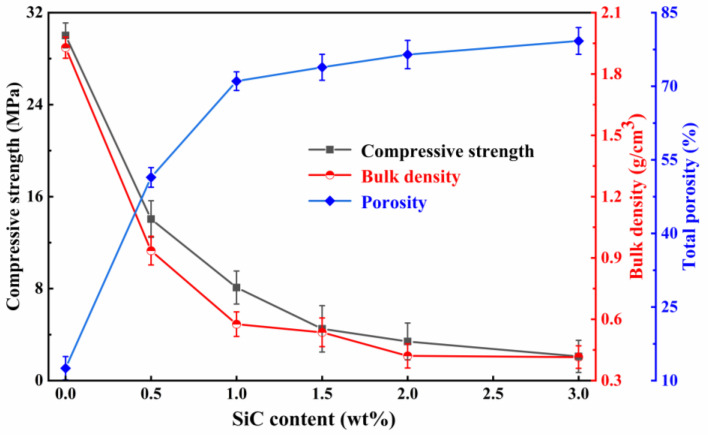
Effects of different SiC contents on the compressive strength, bulk density, and total porosity of the samples.

The content of the foaming agent is one of the key factors in determining the foaming effects of the sample. Figure 11 reveals the effects of various SiC additions on the characteristics of the foamed ceramics when adding 20 wt% SMS and sintering at 1100 °C. When SiC is added from 0 wt% to 1.0 wt%, the bulk density and compressive strength decrease sharply from 1.92 g/cm^3^ and 30.02 MPa to 0.57 g/cm^3^ and 8.09 MPa, respectively. The total porosity increases significantly, from 12.52% to 71.04%. This can be attributed to the fact that SiC is easily oxidized at high temperatures to produce large amounts of gases, which facilitates the formation of porous structures, leading to significant changes in bulk density, compressive strength, and total porosity. Subsequently, the SiC content increases to greater than 1.0 wt%, and the compressive strength decreases. This phenomenon may be due to increased gas production, as more SiC is involved in the oxidation reaction. The increased internal pressure of the generated gas causes the bubbles to break, fuse, and form defective pores, increasing the open porosity. However, when the SiC addition exceeds 1.0 wt%, minimal disparities in bulk density values are observed compared to the samples with a 1.0 wt% addition. This trend highlights the necessity of a moderate SiC addition for achieving sample foaming. When SiC reaches up to 3.0 wt%, the bulk density and compressive strength of the foamed ceramics reach a minimum, while the total porosity attains its maximum value. However, since SiC is a valuable industrial raw material used in producing refractory materials, semiconductor materials, etc., an increase in SiC concentration can dramatically raise the expense of foamed ceramics. Therefore, the initial amount of SiC addition was determined to be 1.0 wt%.

#### 3.3.2. Effects of Foaming Agent Content on Pore Structure

**Figure 12:**
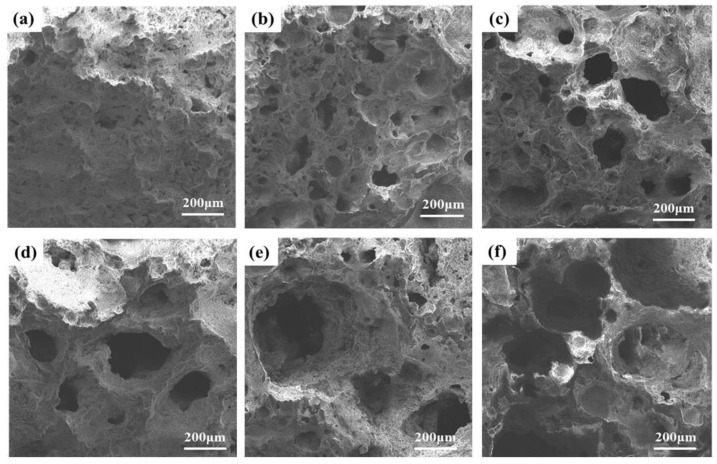
Pore morphology of samples with different SiC contents. (**a**) 0 wt%, (**b**) 0.5 wt%, (**c**) 1.0 wt%, (**d**) 1.5 wt%, (**e**) 2.0 wt%, (**f**) 3.0 wt%.

Figure 12 illustrates the pore morphology of the samples with different SiC contents. Delicate pores were observed in the specimen without SiC addition, shown in Figure 12a. This can be attributed to the presence of air in the molten liquid. It is also possible that the reactions of some elements within the sample released gases. Nevertheless, the tiny pores in the molten liquid could not grow sufficiently due to the insufficient content of the foaming agent. Figure 12b–d reveals that with the introduction of SiC from 0.5 wt% to 1.5 wt%, the number of pores and pore sizes increase significantly. There are two possible explanations for this phenomenon. On the one hand, this may be due to the reduced viscosity of the molten liquid phase. According to Equation (6), the bubble radius grows as the liquid phase viscosity decreases, while the other parameters remain constant [49]. In addition, an increasing amount of SiC is involved in the oxidation reaction to produce gas. The pressure inside the bubble will rise if there is too much SiC present. When the surface tension is less than the pressure gradient inside of and exterior to the bubble, the bubble will rupture and fuse, forming defective pores and inhibiting the development of compressive strength. The pore equilibrium condition can be described by Equation (7) [50,51]:

(7) $$\Delta P=P_{g}-P_{o}$$

where ΔP is the difference between the inner and outer pressure of the bubble, $P_{g}$ is the inner pressure, and $P_{o}$ is the outer pressure of the bubble. The amount of SiC increases the air pressure difference between the inside and outside of the bubble, which also causes a rise in the number of pores, a thinned pore wall, and a drop in the compressive strength of the specimens. Therefore, the optimal SiC addition amount is 1.0 wt% from the perspective of pore structure, mechanical properties, and economic value.

### 3.4. Foaming Mechanism

**Figure 13:**
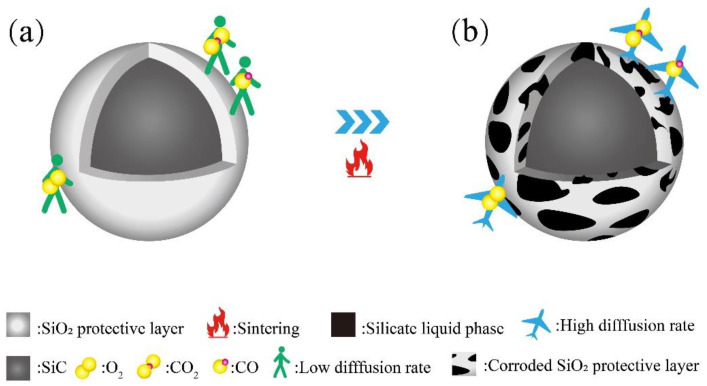
Schematic diagram of the oxidation process of SiC. (**a**) at low temperatures (**b**) at high temperatures.

**Figure 14:**
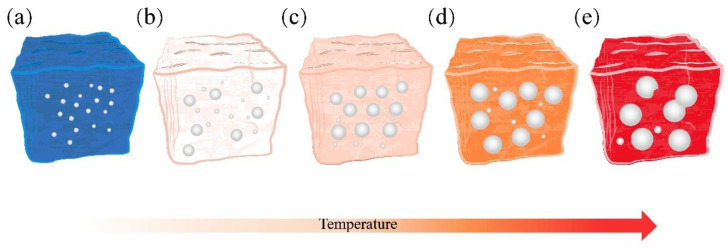
Schematic diagram of the bubble change process. (**a**) 1050 °C, (**b**) 1080 °C, (**c**) 1110 °C, (**d**) 1140 °C, (**e**) 1170 °C.

Figure 13 provides a concise overview of the SiC reaction process. As depicted in Figure 13a, at lower temperatures, SiC particles develop a dense protective SiO_2_ layer on their surfaces, effectively inhibiting O_2_ diffusion into the interior. SiC reacts with O_2_ to produce gas only when O_2_ permeates through the SiO_2_ protective layer. At lower temperatures, the rate of O_2_ permeation through the SiO_2_ protective layer is exceedingly low; only 10^−14^–10^−15^ cm^2^·s^−1^ [21,51]. In Figure 14a, it can be observed that the bubbles within the structure are underdeveloped and dispersed.

As illustrated in Figure 13b, the SiO_2_ protective layer is susceptible to corrosion when exposed to alkaline molten salts at elevated temperatures. This leads to the formation of numerous silicate liquid phases and an accelerated O_2_ diffusion rate, which in turn promotes the reaction between SiC and O_2_, resulting in the production of a significant quantity of CO and CO_2_ gases [52,53]. Due to the increased amount of the liquid phase at high temperatures, the resulting gas stays in the liquid phase and causes the sample to foam. On the other hand, when the SiO_2_ protective layer is compromised, it allows more SiC particles to participate in the reaction, increasing gas generation and facilitating the foaming process. As shown in Figure 14b,c, the sintering temperature is increased from 1080 °C to 1110 °C, and the pores inside the samples are gradually increased and uniformly distributed. When the temperature reaches 1170 °C, the effective viscosity of the liquid decreases to a minimum. The gas trap in the liquid phase applies pressure to the pore wall, which cannot sustain the air pressure within the pore. This results in the formation of connected pores and defective pores, as shown in Figure 14e. The oxidation reaction of SiC during the foaming process is as follows:

(8) $$\mathrm{SiC}+1.5O_{2}(g)={\mathrm{SiO}}_{2}+\mathrm{CO}(g)$$

(9) $$\mathrm{SiC}+2O_{2}(g)={\mathrm{SiO}}_{2}+{\mathrm{CO}}_{2}\left(g\right)$$

According to the above analysis, the sintering temperature and the diffusion rate of O_2_ play an essential role in the foaming process of SiC.

## 4. Conclusions

In this study, the foamed ceramics were successfully prepared using SMS and FA as raw materials using a high-temperature foaming method. The influence of the SMS content, SiC content, and sintering temperature on the characteristics and microstructures of the specimens were discussed. Furthermore, the foaming mechanisms were further analyzed. The main results can be summarized as follows:

1. At a 20% SMS content, both the total and closed porosity peaks of the foamed ceramics promoted the formation of the porous structure. Simultaneously, their compressive strength and bulk density reached 8.09 MPa and 0.57 g/cm^3^, near the foamed ceramic partition board (T/CBCSA 12-2019 [54]) 500-density requirement (0.48 g/cm^3^ ≤ Bulk density ≤ 0.54 g/cm^3^, compressive strength ≥ 7 MPa). Nevertheless, when the SMS content exceeded 20%, the porosity of the foamed ceramics decreased. This was primarily due to a reduced liquid phase content within the structure as the anorthite phase increased. The decrease in the liquid phase content constrained the foaming process and resulted in reduced porosity.
2. In the 1050–1110 °C temperature range, the glassy liquid phase formed by quartz melting corroded the protective SiO_2_ layer on the SiC surface. This accelerated the reaction rate between O_2_ and SiC, trapping the resulting gas in the liquid phase. Consequently, this lowered the foamed ceramics’ bulk density and compressive strength while increasing their porosity. However, the liquid viscosity became insufficient when the firing temperature surpassed 1100 °C. This led to the merging and bridging of bubbles, an increase in the number of connecting holes, and the development of a porous collapsed structure. This was detrimental to the formation of the desired porous structure.
3. At a concentration of SiC of 1.0 wt%, during high-temperature sintering, SiC oxidation released numerous gases that remained in the liquid glass phase, increasing porosity and facilitating foamed ceramics formation. However, when the SiC content exceeded 1.0 wt%, the increased SiC particles in the oxidation reaction generated more gas, leading to elevated pressure within the bubbles. When the surface tension was less than the pressure gradient inside of and exterior to the bubble, the bubble will rupture and fuse, forming defective pores and inhibiting the development of compressive strength. Therefore, considering pore structure, mechanical properties, and economic value, a SiC content of 1.0 wt% is optimal.
4. The best overall performance was achieved using 20 wt% SMS and 80 wt% FA as raw materials, with SiC addition of 1.0 wt% and a sintering temperature of 1100 °C. Under these conditions, the compressive strength, bulk density, and total porosity of the product were 8.09 MPa, 0.57 g/cm^3^, and 71.04%, respectively. Its excellent porous structure and mechanical properties make it suitable for use as an insulating material or as a decorative material for building partitions.

## Figures and Tables

**Table 1:** Chemical composition (wt%) of fly ash and silicomanganese slag.

Raw Materials	SiO_2_	Al_2_O_3_	CaO	MgO	Fe_2_O_3_	Na_2_O	K_2_O	MnO	SO_3_	TiO_2_	Others
FA	57.01	30.57	2.36	1.44	4.36	1.71	0.38	0.04	0.57	0.79	0.67
SMS	42.02	13.49	26.38	5.00	0.48	0.74	1.85	7.53	0.75	0.87	0.89

**Table 2:** Raw material compositions of the samples.

Sample	Temperature (°C)	FA/%	SMS/%	SiC/%	Na_2_B_4_O_5_(OH)·10H_2_O/%	Bentonite/%
A1	1080 °C	100	0	0.5	10	5
A2	1080 °C	90	10	0.5	10	5
A3	1080 °C	80	20	0.5	10	5
A4	1080 °C	70	30	0.5	10	5
A5	1080 °C	60	40	0.5	10	5
A6	1080 °C	50	50	0.5	10	5
A3-1	1050 °C	80	20	0.5	10	5
A3-2	1080 °C	80	20	0.5	10	5
A3-3	1110 °C	80	20	0.5	10	5
A3-4	1140 °C	80	20	0.5	10	5
A3-5	1170 °C	80	20	0.5	10	5
A3-3-1	1110 °C	80	20	0	10	5
A3-3-2	1110 °C	80	20	1	10	5
A3-3-3	1110 °C	80	20	1.5	10	5
A3-3-4	1110 °C	80	20	2	10	5
A3-3-5	1110 °C	80	20	3	10	5

## Data Availability

All relevant data generated by the authors or analyzed during the study are included within the paper.
